# Toward Relative Redox Potential Predictions in Flavoproteins: Treatment of a Charge-Changing Mutation in Alchemical Free Energy Simulations

**DOI:** 10.1021/acs.jctc.6c01142

**Published:** 2026-08-13

**Authors:** Sarah Elhajj, Jacopo D’Ascenzi, Massimo Olivucci, Samer Gozem

**Affiliations:** † Department of Chemistry, 1373 Georgia State University, Atlanta, Georgia 30302, United States; ‡ Dipartimento di Biotecnologie, Chimica e Farmacia, 9313 Universita di Siena, Via A. Moro 2, 53100 Siena, Italy; § Chemistry Department, Bowling Green State University, Bowling Green, Ohio 43403, United States

## Abstract

Redox enzymes play an essential role in nature and in biotechnological applications such as (photo)­biocatalysis and biosensing. Understanding how a protein’s sequence and structure tune its redox potential is very valuable for engineering proteins with tailored (photo)­redox properties. Since protein redox potential measurements are laborious, reliable redox potential computations present an attractive alternative. However, redox calculations come with their own set of challenges, such as ensuring adequate sampling and accurate force fields. Dealing with charge-changing states introduces additional challenges to theory and requires special considerations in the model setup. Here, we report a protocol for computing the change in the proton-coupled one-electron redox potential associated with a D63N charge-changing mutation in a prototypal flavoprotein, *Desulfovibrio vulgaris* flavodoxin. An automated average protein electrostatic configuration protocol, APEC-F 2.0, was used to construct hybrid quantum mechanics/molecular mechanics (QM/MM) models. These models were used for subsequent alchemical free energy simulations in which a charged surface aspartate residue was gradually converted to an isosteric but neutral asparagine (D63N) over 40 λ windows. A thermodynamic cycle was employed to calculate the redox potential of the mutant relative to the wild-type reference. This calculation was repeated for the same D63N mutation using models prepared under slightly different conditions, focusing primarily on factors that affect the electrostatic environment in the system. Factors tested include (1) the effect of including extra salt ions in the model solution, (2) different protocols to balance the disappearing negative charge associated with the alchemical D63N mutation, and (3) the effect of accounting for the kinetic energy terms in the free energy protocol due to alchemical morphing of the atomic masses. The results indicate that such apparently minor details may have a considerable effect on the random and systematic errors obtained from the free energy simulations. The best models were shown to reproduce the experimental shift in the redox potential due to the D63N mutation with an accuracy of 0.3 kcal/mol. The associated error for this shift is 1.3 kcal/mol, calculated as the total standard deviation of triplicate simulations for each of the oxidized and reduced neutral semiquinone states of flavin. The addition of salt ions in the simulations and the proper treatment of charge-conserving coalchemical counterions in particular are found to be paramount, since less accurate models led to errors more than double the magnitude compared to the best protocol. The sources of those errors are discussed and often found to be associated with medium-range electrostatics due to missing or inaccurate second solvation/ionic shells around the mutation site.

## Graphical abstract

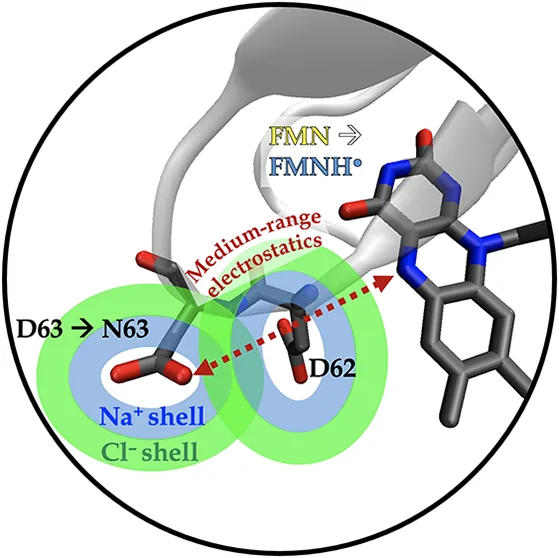

## Introduction

Redox reactions are ubiquitous in chemistry and nature. For many reactions, natural and artificially designed redox enzymes remain the only feasible catalysts that provide a direct synthetic route, with no traditional (non biocatalytic) counterpart available.^1,2^

In light of rapidly advancing developments in flavin photocatalysis, there is a strong incentive to understand how to tune flavin’s excited state redox potentials.^3–11^ Flavin’s excited-state redox potentials are also relevant to its role in electron transfer processes in several classes of blue-light photoreceptors such as LOV, BLUF, Cryptochromes, and DNA photolyases,^12–26^ and to its role as a natural photosensitizer.^27–29^

Computational modeling can provide valuable insight into the principles of (photo)­biocatalyst design.^30,31^ Excited-state reduction potentials (*E*_red_^*0^) can be derived from first-principles by calculating the ground state reduction potential (*E*_red_^0^) and adiabatic excitation energy (*E* ^00^) separately, as shown in eq 1 below and in the thermodynamic cycle shown in Figure 1.

1 $$E_{\mathrm{red}}^{*0}=E_{\mathrm{red}}^{0}+E^{00}$$

**Figure 1:**
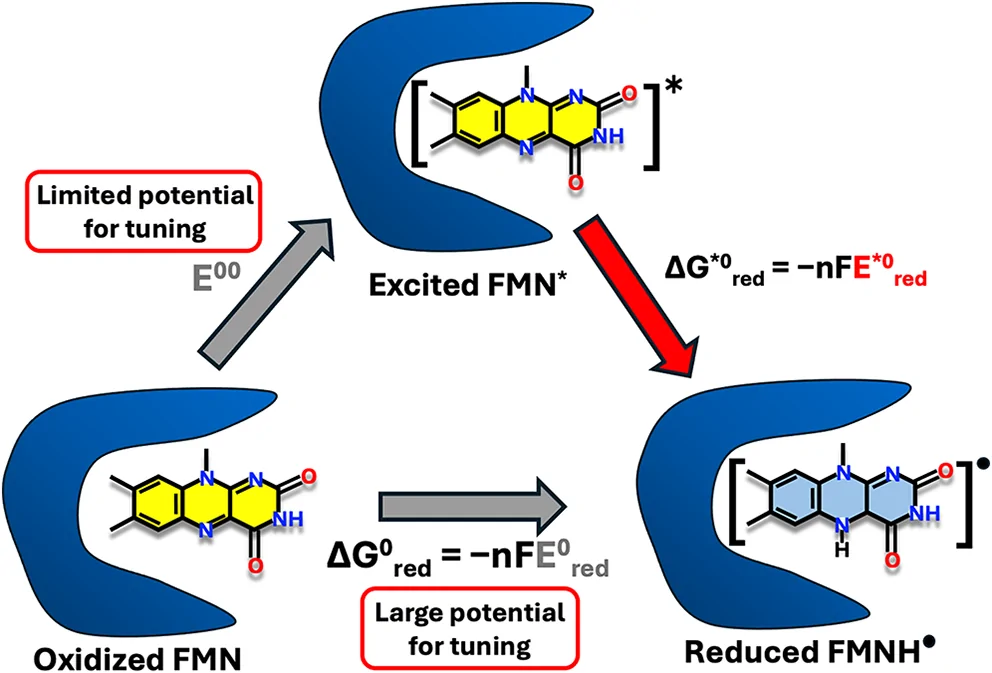
A thermodynamic cycle showing how the excited state reduction potential, *E*_red_^*0^, is expressed as the sum of the ground state reduction potential *E*_red_^0^ and the 0–0 adiabatic excitation energy *E* ^00^. *E* ^00^ represents the energy difference between the ground state and excited state energies at their respective minima, corrected for the difference in zero-point vibrational energy (ZPVE). *n* refers to the number of electrons transferred during the redox reaction, and F is Faraday’s constant. To maintain the same units in the thermodynamic cycle, a suitable unit for F should be used. E.g., if *E* ^00^ is in units of eV, then it is suitable to simply use the negative of the redox potential *E*_red_^0^ in Volts multiplied by n to yield eV (*F* = 1 eV/V), while if *E* ^00^ is in kcal/mol then Δ*G*_red_^0^ can be calculated using the equations shown in the figure with (*F* = 23.061 kcal/mol·V). The proton-coupled one-electron reduction of protein-bound flavin is used as a representative example here.

There are two approaches to tune photoredox potentials in flavoproteins. One is through the use of chemically modified flavin analogues such as roseoflavin, deazalfavins, and deazaalloxazines, which can control and expand the absorption/fluorescence spectral range and redox potential through substitutions at various positions on the isoalloxazine ring.^32–42^ The second approach is to tune the photoredox potential of the native FMN or FAD cofactor through protein sequence mutations. The latter approach is often more tractable, especially for *in vivo* applications.

Of the two ingredients in eq 1, the ground state reduction potential *E*_red_ is far more tunable than *E* ^00^. Numerous experimental^43–49^ and computational^50–57^ spectral tuning studies on flavin have shown that it is difficult to modify flavin’s absorption wavelength by more than 10–20 nm (this is equivalent to ∼0.1 eV which is on the order of ∼100 meV energy or 100 mV potential for a 1-electron process). Meanwhile, flavin’s redox potential can be tuned over **hundreds** of mVs (on the order of ∼500 mV depending on the protein).^33,58–67^ Therefore, tuning flavin’s excited state reduction potential, *E*_red_^*0^, can primarily be achieved by modifying the ground state reduction potential, *E*_red_^0^.

While we present ground state redox potentials as an ingredient of excited state redox potentials above, flavin’s ground state redox properties are key to its versatility as an enzyme cofactor. Accurate ground state redox potential predictions in flavoproteins have important applications to a wide range of problems in biocatalysis and electron bifurcating systems.^68–72^

There are well established computational methods for simulating excitation energies for flavins.^73–75^ There are also several well-documented and well-tested methods for simulating redox potentials of molecules in solution.^76–82^ However, there are far fewer established computational protocols for redox simulations of protein-bound systems like flavoproteins. Therefore, it is important to revisit the simulation of ground state redox potentials, especially due to the wealth of experimental reference data for ground state redox potentials that can be used as a reference. If ground state redox potentials can be accurately predicted, then excited state redox potentials can also be predicted with similar accuracy when using methods that accurately reproduce *E* ^00^.

Of the many enzymes and photoreceptors that bind flavin, flavodoxin is among the best characterized with regards to its redox properties. Redox measurements have been reported in a range of wild-type and mutant flavodoxins from organisms including *Clostridium beijerinckii*, *Desulfovibrio vulgaris*, and *Anabaena*.^83–86^ Such measurements serve as a useful reference for computational modeling of redox potentials in flavoproteins.^83–85^ The successful simulation of redox potentials in a well characterized system like flavodoxin will pave the path toward simulating other systems where the flavin is not electrode-accessible or where excited state redox potentials are needed.

In the next section, we review existing work on flavin ground state redox simulations. We then describe the approach used in this work, which builds on a recently reported Average Protein Electrostatic Configuration for flavoproteins (APEC-F 2.0) QM/MM approach^73^ as a starting point for free energy simulations using a thermodynamic cycle analogous to those used in relative binding free energy (RBFE) simulations. Here we refer to this as the relative redox potential (RRP) approach, as shown in the cycle within the magenta box in Figure 2. We then present and discuss the results of the calculations for models set up using different conditions.

**Figure 2:**
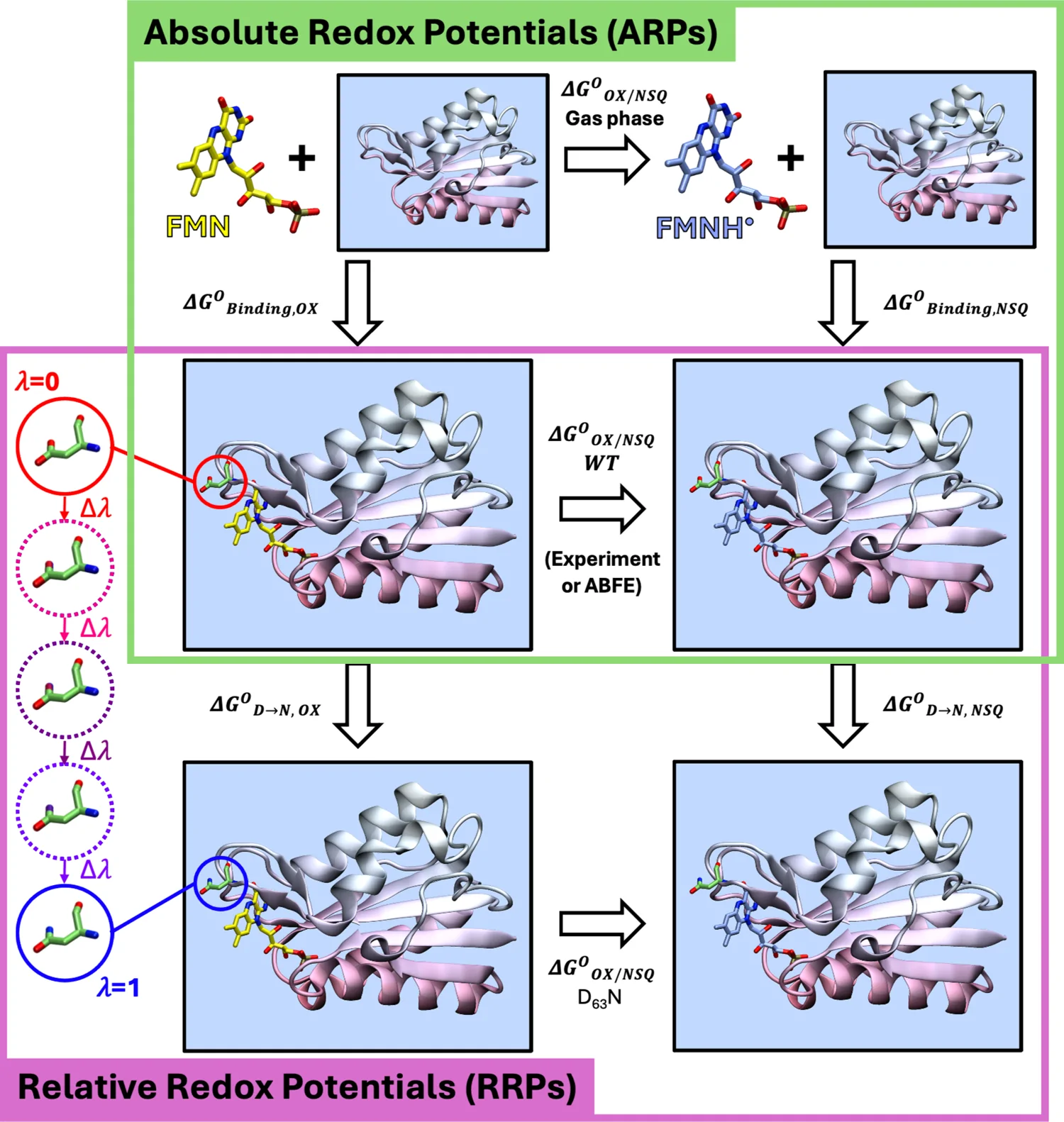
Schematic representation of an alchemical free energy approach for simulating redox potentials. The bottom four panels (magenta border) represent the thermodynamic cycle used to calculate the relative redox potentials (RRPs) for a flavodoxin mutant compared to the wild type. The bottom horizontal leg represents the desired reduction free energy of the mutant (Δ*G*_OX/NSQ_(*D*_63_ *N*)). The vertical legs represent the alchemical transformation of an amino acid side chain (in this case, from the wild-type D63 to N) for flavin in the oxidized (OX) and reduced neutral semiquinone (NSQ) states. The fourth leg in that cycle is the free energy associated with reducing the flavin in the wild-type flavodoxin (Δ*G*_OX/NSQ_(*WT*)). This free energy can be obtained from an experimental reference, as done in this work, or by using an additional thermodynamic cycle (green border) to calculate the absolute redox potentials (ARPs). The ARPs would require a method that accurately captures the binding free energy of FMN and FMNH^•^ state to the flaxodoxin apoprotein, and are not attempted in this work.

## Methodology: Background

Free energy simulations are widely used to determine properties such as free energy of solvation^87–92^ or protein–ligand binding energies.^93–98^ Numerous studies have shown that it is possible to predict such free energies using alchemical simulations to an accuracy within 1 kcal/mol.^99,100^ Redox simulations pose a unique challenge to free energy simulations because the reduction involves a change in charge and/or protonation of the cofactor. Therefore, while the success of former free energy simulations is encouraging, redox potential simulations may require extra care.

Redox free energy simulations in proteins are often carried out within the framework of the linear response approximation (LRA)^4,101–103^ or using methods related to free energy perturbation (FEP).^104–107^ The simulations may be performed using classical (MM), semiempirical, quantum mechanical (QM), or hybrid (QM/MM) potentials.^108^ Classical MM force fields are the most widely used due to their affordability, while QM or QM/MM can introduce higher accuracy^109^ albeit at a potentially much higher computational cost. QM/MM redox potential predictions are typically only used with the LRA either with electrostatic embedding scheme or by accounting for polarization, e.g., using a polarizable force field or Effective Fragment Potential (BioEFP) approach.^4,103,110^

Relative redox potential calculations in flavodoxins have only been performed using classical MM free energy simulations to the best of our knowledge. Satelle and Sutcliffe relied on a thermodynamic integration (TI) formalism^104,111^ to calculate the changes in redox potential of a wild type *Anabaena* flavodoxin in a series of six single-point neutral mutations.^112^ They correctly reproduced the experimental trend for the effect of each mutation in a protocol comprising 14 discrete molecular dynamics (MD) production runs, each sampled for 1.5 ns. Their computed potentials vary from the actual redox potential by 20–100 mV (∼0.5–2.3 kcal/mol). However, their study focused solely on neutral mutations. In a more recent study, Vertemara and co-workers^113^ adopted a nonequilibrium TI approach^114–116^ to compute redox potentials of eight single-residue mutants of *wt*-*Clostridium beijerinckii* flavodoxin. 75% of their predicted values were within 1 kcal/mol of the experimental values.^113^ Again, most of their mutations involved neutral residues. Calculations by Ishikita^117,118^ on *Desulfovibrio vulgaris* flavodoxin, as well as on *wt*-*Clostridium beijerinckii* and its mutants, employed an implicit continuum model to describe the electrostatic environment of the protein, while accounting for charges from titratable sites in the protein. The electrostatic environment was created by solving the linear Poisson–Boltzmann equation with MEAD^119^ to model the redox potential relative to free flavin in solution.

Although the aforementioned computational studies on flavodoxin^112,113^ used different approaches, they instill confidence that classical force fields can give reasonable trends in redox potential. However, this does require that the redox site be carefully parametrized in both redox states to give a realistic description of flavin’s interactions with the protein.

Since there is no clear recipe for parametrizing flavin’s redox states (or its excited states for photoredox potentials), we take a different approach here where we focus on obtaining the flavin’s electronic structure and charges from a QM/MM protocol, APEC-F 2.0.^73,120^ We combine APEC-F 2.0 with the RRP thermodynamic cycle (Figure 2) to compute the redox potential for a D63N charged mutation in *Desulfovibrio vulgaris* flavodoxin. The goal is to test the effect of a size-conserving perturbation that is accompanied by a different charge.

The main focus of this work is to introduce the hybrid APEC-RRP approach to calculate the relative free energies associated with flavoprotein mutations. The second main goal, in view of existing guidelines on free energy calculations involving charge-changing mutations,^121^ is to test the effect of different treatments of solution ions and counterions on the redox potential calculations.

## Computational Methods

### Choice of the Model System

Previous studies simulating redox potentials have focused mainly on flavodoxins from *Anabaena* ^112^ (PDB ID: 1FLV) and *Clostridium beijerinckii* ^113^ (PDB ID 5NLL and 2FOX for oxidized and neutral-semiquinone states, respectively). Here we compute the redox potential for the oxidized (OX) state to the neutral semiquinone (NSQ) state of flavodoxin from *Desulfovibrio vulgaris*. There are two main reasons for this choice. The first reason is the availability of a well-resolved (1.35 Å) low-temperature X-ray crystal structure PDB ID 1J8Q. The second reason is the availability of experimental studies on *Desulfovibrio vulgaris* flavodoxin that have systematically investigated the effect of charged mutations near flavin.^84^ We focus on one mutation in particular, D63N, which has the largest effect on the *E*_OX/NSQ_ potential due to its proximity to flavin.^84^ *E*_OX/NSQ_ is referred to as E2 in the experimental literature and corresponds to the proton-coupled one-electron reduction of FMN to FMNH^•^.

In reference to D63N, we adopt the residue numbering used by Swenson and co-workers (D63N) and consistent with the Swiss-Prot sequence.^84^ However, this residue appears in PDB ID 1J8Q as residue number 62. This is because the crystallographers could only isolate and crystallize a protein where the first two residues (Met-Pro) are replaced by Alanine (Ala). Swenson and co-workers used the alanine variant in their experiments instead of the Met-Pro protein.^84^ This means that the crystal structure we are using is consistent with the protein used in the corresponding experiment.

### QM/MM Free-Energy Minimized Structure Powered by APEC-F 2.0

MD simulations normally require careful parametrization of the cofactor in both its oxidized OX and reduced neutral semiquinone NSQ states. Here, we opt for an alternative approach; we build the free energy protocol on top of QM/MM optimized FMN and FMNH^•^ geometries and charges, which are kept frozen during subsequent dynamics. The charges are derived from QM/MM calculations using the ElectroStatic Potential Fitted (ESPF) approach.^122^ As discussed in ref 73, the geometrical rigidity of the flavin redox-active isoalloxazine rings justifies the use of such an approximation. The full details of the QM/MM protocol, which was automated as part of the APEC-F 2.0 package, are provided in the same reference.

The APEC-F 2.0 QM/MM flavodoxin models in the OX and NSQ states were generated in a 9 × 9 × 9 nm^3^ cubic box containing over 23,000 TIP3P water molecules. Wild-type flavodoxin has many ionizable residues, all of which are on the protein surface (see Figure 3A). The negatively charged residues (ASP and GLU, shown in red in Figure 3A) substantially outnumber the positively charged residues (HIS, ARG, LYS, shown in blue). We assume in our models that all those amino acids are in their standard charged state (including histidine). We also assume that the FMN’s phosphate group is fully deprotonated and therefore in the −2 charge state, resulting in a flavodoxin total charge of −20.^123^ Therefore, we generated two sets of models. One set included a minimal number of Na^+^ counterions needed to neutralize the protein. This is labeled as the minimal NaCl (0 mM NaCl) model, and only has 20 Na^+^ ions (see Figure 3C). A second set of QM/MM constructed models included additional NaCl ions and is labeled as the extra NaCl (∼100 mM) model. This model has 62 Na^+^ ions and 42 Cl^–^ ions in total (see Figure 3D).

**Figure 3:**
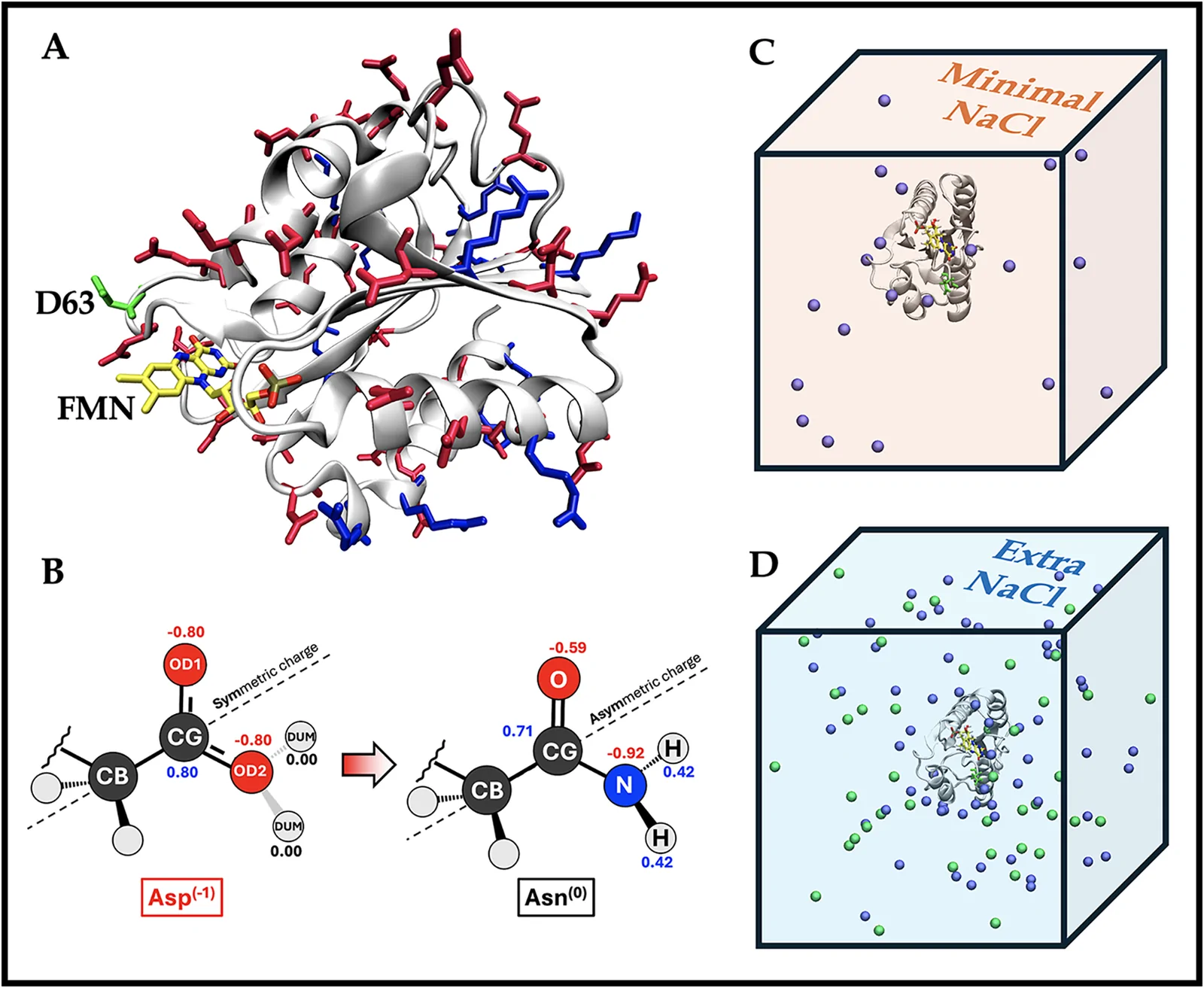
(A) Structure of wild type *D. vulgaris* flavodoxin with all titratable amino acids shown. 17 ASP and 13 GLU amino acids are shown in red (or, in the case of D63, in green), while 1 HIS, 4 LYS, and 7 ARG amino acids are shown in blue. In this model, the protein secondary structure elements are represented in silver and flavin as licorice in yellow. (B) The change in charges on some of the D63 side chain atoms upon alchemical transformation to an asparagine. (C, D) A snapshot of models having minimal NaCl and extra NaCl, respectively. Na^+^ are shown as blue spheres and Cl^–^ as green spheres. Panels (A, C, and D) were prepared using vmd.^124^

### Molecular Dynamics Setup and Equilibration

Starting from an optimized free energy structure generated by the APEC-F 2.0 protocol,^73^ MD simulations were performed for the OX and NSQ models while keeping the flavin cofactor frozen in its optimized QM/MM geometry. Applying constraints on the FMN and FMNH^•^ geometries comes with an entropic penalty for restricting the translational, rotational, and vibrational degrees of freedom. However, we assume that those entropy terms would largely cancel out in the two vertical legs of the RRP thermodynamic cycle shown in Figure 2.

The MD protocol adopted here for free energy calculations employs similar settings and parameters as used in APEC-F 2.0 to maintain consistency of two protocols. Namely, we adopted the GROMACS 2024.4 MD software^125^ and the amber99sb force field.^126^ Bonds involving hydrogen atoms were constrained using LINCS algorithm^127,128^ and a time step of 2 fs was used. Long-range electrostatics were treated with particle mesh Ewald (PME)^129,130^ with a real space cutoff of 0.8 nm, a Fourier grid spacing of 0.12 nm, a PME order of 4, and relative strength at the cutoff of 10^–5^. The van der Waals interactions were cut off at 0.8 nm. Dispersion corrections were applied for energy and pressure.

### Alchemical Free Energy Calculations

To prepare gro and top files needed for the mutation free energy calculations, the pmx Python library^131,132^ was used. In pmx, the amber99sbmut force field incorporates dummy atoms in a hybrid topology fashion.^132–135^ Dummy H atoms were renamed to be detected by the GROMACS LINCS algorithm as hydrogens. Specifically, all DH labels in atom names were converted to HD, as well as DUM_H to H_DUM.

The side-chain atoms of the mutated amino acid (D63) were treated with soft core potentials for the coulomb and Lennard-Jones interactions.^136^ The following settings were used in association with the soft-core potential: sc-α = 0.5, sc-coul = yes, sc-power = 1, sc-sigma = 0.3. Those parameters are consistent with those used in the GROMACS tutorial on solvation free energy of methane, which we then tailored to our needs to deal with a larger and charge-changing mutation.^137–139^ The couple-intramol parameter was set to yes, as the initial and final states connected by a free energy RRP calculation are two different proteins (WT vs mutant), and the internal energy of the mutated group is thus a contribution that needs to be accounted for.

In several of our tested protocols, the gradual disappearance of the charged amino acid was balanced by gradual morphing of an appropriate buffer ion, requiring the inclusion of the latter in the hybrid topology (see the following section for more details).

A typical MD protocol involves equilibration steps using both NPT and NVT dynamics. However, the freezing of the flavin cofactor in its QM/MM optimized geometry is not compatible with the NPT coordinate rescaling algorithm, as discussed in prior work.^73^ Therefore, the equilibrated volume is determined at the level of APEC-F 2.0 using NPT with a flexible chromophore before performing all dynamics and free energy simulations using canonical (NVT) dynamics, following the steps of De Vivo and co-workers.^140^

Keeping the frozen chromophore approximation adopted in APEC, the starting zero-th λ window is initiated with a 300 ps heating step from 0 to 298 K followed by a 3 ns (or, in some test protocols, 50 ns) equilibration. All the following λ windows were initiated from the last configuration sampled in the previous step’s 3 ns equilibration.

Alchemical free energy calculations were performed using an equilibrium approach.^140–143^ A total of 14 different protocols were tested for calculating the redox potential associated with the D63N mutation in *D. vulgaris* flavodoxin. The details of those tests are described more fully at the start of the Results and Discussion section.

For each protocol tested, we employed the same RRP thermodynamic cycle reported in Figure 2 to obtain the relative reduction potential. The reported potentials for each protocol are based on the average and standard deviation of a triplicate of replicas for each of the OX and NSQ states.^144^ A replica herein refers to a simulation of an identical system and conditions, but initiated with different randomized velocities. We adopted three replicas consistent with guidelines suggested by Coveney and Soares and co-workers.^145,146^ For each replica, the alchemical path was stratified into 20 or 40 discrete λ windows, each comprising a 3 ns equilibration and a 12 ns production run. For the 40 λ windows, the transformation was carried out at (λ = **0**, 0.002305, 0.00461, **0.00922**, 0.01422, 0.01922, 0.03358, **0.04794**, 0.06294, 0.07794, 0.096495, **0.11505**, 0.13505, 0.15505, 0.180695, **0.20634**, 0.26121, **0.31608**, 0.37673, **0.43738**, 0.5, **0.56262**, 0.62327, **0.68392**, 0.73879, **0.79366**, 0.819305, **0.84495**, 0.86495, 0.88495, 0.903505, 0.92206, 0.93706, **0.95206**, 0.96642, **0.98078**, 0.98578, 0.99078, 0.99539, **1**). Our series of λ windows builds on the 14 Gaussian distributed λ values (highlighted in bold) that Section 25.1 of the AMBER manual suggests, with the addition of more intermediate windows to improve accuracy. The addition of λ steps focuses on introducing narrower windows at the extremities but larger windows in the middle of the λ path. We also adopted a sequential equilibration protocol as suggested in Section 39.6 of the 2024 Amber manual.^147^ In this approach, only the equilibration steps were serialized, such that the calculations can be run in a staggered fashion. In other words, the last frame of the equilibration at one λ step served as a starting point for the production MD for that step, while also serving as a starting point for the equilibration of the next λ step. All MD simulations were performed on the ARCTIC facility using nodes equipped with NVIDIA Tesla V100 GPUs.

The D63N alchemical transformation of negatively charged aspartic acid to neutral asparagine modifies the total charge of the system.^121^ Therefore, the computations must account for a counterion to prevent the rise of any net charge in the simulation box during the mutation process. This can be done using a universal background charge, or by morphing an explicit counterion.^121,140,142^ In the universal background charge approach, the particle mesh Ewald (PME) algorithm neutralizes the box via the addition of an implicit background homogeneous charge.^148^ Therefore, we refer to this background charge approach throughout this work simply as the default PME option.

To illustrate the second approach, as a negative charge disappears (D63N), we concomitantly converted a sodium ion Na^+^ into a water H_2_O molecule. We label this approach NAB. We also tested the alternative approach of converting a water molecule (neutral) into a chloride ion Cl^–^, which we label as BCL. The charge of this counterion (NAB or BCL) was coperturbed with the mutant along the λ path to keep the net charge of the system constant throughout all λ steps. Further testing was performed to investigate the effect of allowing the BCL or NAB ions to move as compared to fixing their position. When fixed, the coalchemical ions were kept near the edge of the solvent box several nanometers away from the mutant site. Given the thermodynamic cycle adopted (see Figure 2), the buffer contributions to the free energy of the system should cancel out with each other in the two vertical legs of the cycle. Note that the treatment of the counterion is an independent factor with respect to the ionic strength variation performed in the APEC-QM/MM simulation (minimal vs extra NaCl counterions).

Sample mdp files, along with a script that modifies the gro, top and itp files to alter the labels of dummy hydrogens, are provided (see Data Availability section).

### Analysis Tools

The Δ*G* associated with the D63N mutation for each of the OX and NSQ states was obtained from the corresponding MD simulations using the Bennett Acceptance Ratio (BAR) method implemented in GROMACS’ *gmx bar*.^149^ These two free energies provide the values for the two vertical legs of the RRP thermodynamic cycle in Figure 2. For the third leg, corresponding to the reference potential, we use the experimentally determined reduction potential of wild type *Desulfovibrio vulgaris* flavodoxin, −148 mV.^84^ The necessary unit conversions are then applied to produce the reduction potential of the D63N mutant, which is then compared with the experimentally measured one (−189 mV, a 41 mV decrease in reduction potential relative to the wild type).^84^

While absolute redox potentials (ARPs) can in principle be computed using the top thermodynamic cycle shown with a green border in Figure 2, we do not attempt this in the present work, as we focus on capturing the relative redox potential (RRPs) as accurately as possible in a system where the perturbation (D63N) is relatively small (barring a change in charge).

The *gmx rdf* tool was used to determine the radial distribution function (RDF) of Na^+^ and Cl^–^ ions relative to the mutation site along each λ trajectory, as well as the cumulative number (CN) obtained by integrating the RDF. The CN value represents the average number of particles within a given distance. The total integrated charge difference (Na^+^ – Cl^–^) was calculated from the difference in the coordination number of the Na^+^ and Cl^–^ ions.

### Testing GROMACS 2024.2 and 2024.4

Before the 2024.4 version of GROMACS, *gmx bar* did not account for the effect of mass morphing on the contribution of kinetic energy to the free energy. This kinetic contribution was implemented in GROMACS 2024.4, as discussed in the GROMACS User Discussion forum in ref 150. We elaborate further on this in the SI and in the Results and Discussion section.

## Results and Discussion

When performing free energy simulations, there are many decisions to be made regarding the methodology and optimal parameters to use. Although adequate sampling and reasonable force fields are paramount, other factors in the model setup are also important to consider. Here, we focus on systematically investigating parameters that are especially relevant to the physics of charged mutations: The treatment of counterions and solution ions.

Figure 4 summarizes the main tests carried out in this work, as well as the corresponding computed redox potentials. Overall, there are 14 sets of calculations carried out under slightly different conditions, with each set comprising 6 free energy calculations (3 for the OX state and 3 for the NSQ state). The factors varied across the 14 tests are itemized below:

- The effect of changing the total number of λ windows from 20 to 40.
- The influence of ionic strength (i.e., using minimal counterions or adding extra salt ions) in the setup of the QM/MM models and for subsequent free energy simulations.
- The effect of increasing the equilibration time at the beginning of the free energy workflow (only at λ = 0) from 3 to 50 ns.
- The effect of adopting different buffer molecules (or coalchemical ions) versus using a uniform background charge.
- The effect of fixing the coalchemical ion versus allowing it to move freely in the simulation.
- The effect of the mass morphing implementation added in GROMACS 2024.4 compared to using GROMACS 2024.2.

**Figure 4:**
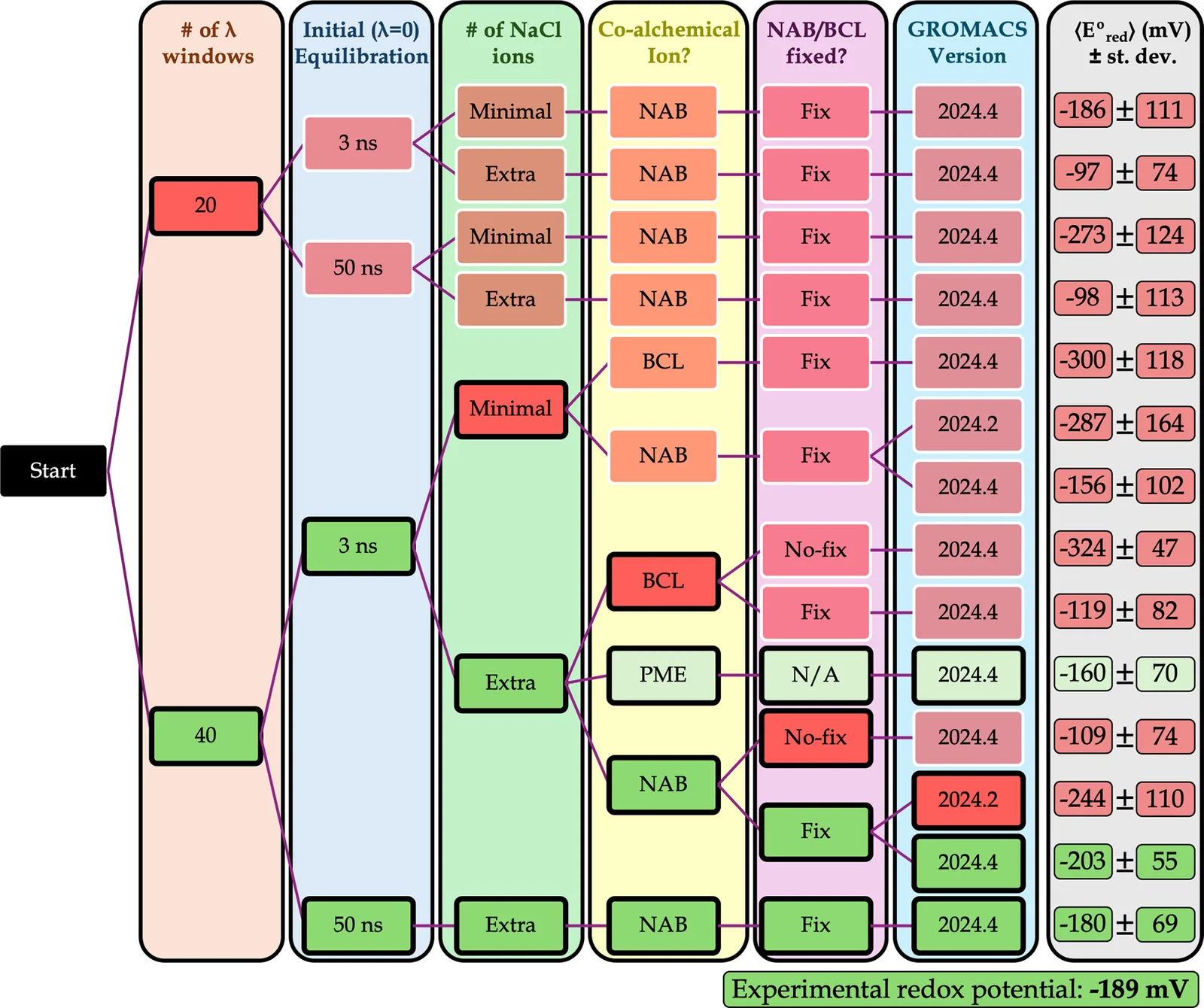
A workflow describing the different tests carried out in this work. The first level is related to the number of λ steps used in the free energy BAR protocol. The second level is related to the length of equilibration carried out before initiating the full free energy protocol (i.e., at the first NVT step of the first λ point). The third level is whether to use only a minimal number of ions to neutralize the charge of the protein without any additional NaCl ions, or to include an additional 42 NaCl ions in solution. The fourth level is concerned with how to balance the disappearing negative charge as the D63N mutation is gradually introduced. **BCL:** A water molecule is coalchemically converted into a chloride ion in solution as the negative charge of the D63 mutant disappears; **NAB:** A sodium ion is coalchemically converted into water as the D63 mutant charge disappears; **PME:** The disappearing negative charge is balanced by a growing negative uniform background charge introduced in the model. The fifth level decision is whether to keep the coalchemical ion fixed near the border of the box or to allow it to move through the solvent. Lastly, the decision about the GROMACS version is related to whether a kinetic energy contribution from mass perturbation of the alchemical ion is included in the simulation (version 2024.4) or not (version 2024.2). The last column shows (in mV) the redox potential computed using each protocol and the standard deviation. The corresponding experimental redox potential obtained from ref 84. is shown at the bottom. Note that the redox potentials are computed from a series of 3 replicas in the OX state and 3 replicas in the NSQ state; the associated error is obtained from the standard deviations from the two sets of triplicate replicas.

Figure 5B plots the average redox potentials from the last column of Figure 4. The error bars indicate the standard deviations obtained from the sum of the variance of 3 replicas of the OX state and 3 replicas of the NSQ state, shown in Figure 5A. The corresponding breakdown for each replica is reported in the Supporting Information document (Table S1). Overall, Figure 5B highlights the gradual convergence of the calculations close to the experimental value as the number of λ windows increases from 20 to 40, as additional solution ions are introduced to the solution (moving from minimal to extra NaCl models), and as the counterion is treated either using a uniform background charge with PME or using the NAB/BCL coalchemical approaches.

**Figure 5:**
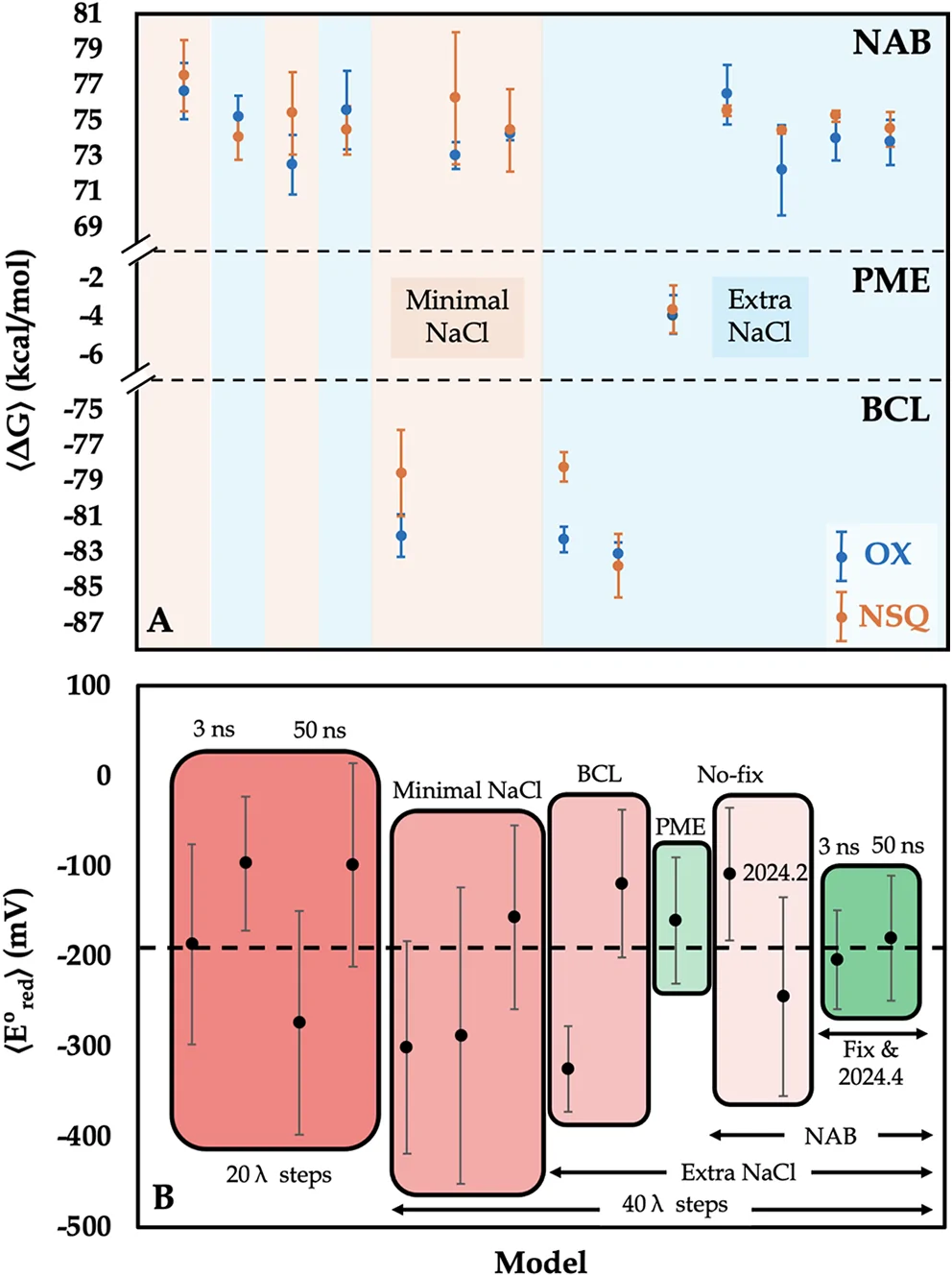
(A) The average free energies (Δ*G*) computed from three replicas associated with the D63N mutation for each of the OX (blue) and NSQ (orange) states for the models presented in Figure 4. The associated standard deviations are shown as error bars. A light blue background indicates models that have extra NaCl ions, while a light orange background indicates models that have minimal NaCl. (B) Average computed redox potentials from the last column of Figure 4. The potentials in panel (B) are obtained from the Δ*G* free energies aligned above it in panel (A). Units of mV are used here instead of kcal/mol. The associated standard deviations (obtained from the OX and NSQ standard deviations) are shown as error bars. The colored boxes indicate how the calculations approach the experimental value (represented as a dashed line) through improvements in the computational protocol. The data from this plot is tabulated in Table S1 of the SI.

Several patterns emerge from these plots. Specifically, using 40 λ windows generally gave more consistent results than using 20 λ windows. Furthermore, the minimal NaCl models resulted in errors larger than those using extra NaCl. To differentiate the two types of models, we use a different color background panel in Figure 5A. Δ*G*s computed using the same conditions but with minimal counterions (orange background) are generally larger than Δ*G*s computed with the extra NaCl model (blue background). This is observed across both the λ = 20 and λ = 40 sets of results. For example, for λ = 20, in the minimal NaCl models, the total standard deviation from 3 replicas in the OX state and 3 replicas in the NSQ state were both large (Figure 5A), totaling over 111 mV for the corresponding redox potentials (Figure 5B). Lastly, we find that the NAB approach and PME both appear to outperform the BCL approach. Using a BCL counterion appears to introduce systematic errors.

Some of those results may be intuitive. For example, alchemical modification of a negatively charged residue (D63) to a neutral one in a minimal ion model that only contains Na^+^ counterions and no Cl^–^ ions can result in a larger perturbation of the overall energy than in a model that contains both ions. However, it is not always obvious why some models introduce larger systematic or random errors than others. Therefore, in the remainder of this section, we focus on understanding the sources of some of those errors.

### Number of λ Windows and Pre-Equilibration Time

The first decision to establish any free energy protocol is the length of the sampling time and number of λ windows needed to ensure that there is sufficient phase space overlap between neighboring windows. It is sometimes recommended to use a split λ approach in which the charge and Lennard–Jones (LJ) interactions are modified separately.^100,151^ However, the D63N mutation involves simply transforming an oxygen atom into an amine (NH_2_) group, which is associated with a relatively small change in the steric and van der Waals interactions. Therefore, we opted to simultaneously scale the charge and LJ parameters together as a function of λ. We expect here that the largest effect will come from the charge modification rather than the LJ potential.

As shown in Figures 4 and 5, 20 λ steps often resulted in errors that were too large to be useful for predicting the change in the redox potential upon introducing the D63N mutation. Even with the addition of extra solution ions and equilibrating the system for a longer time (50 ns), the predicted redox potential was −98 mV, far from the experimental redox potential of −189 mV. Furthermore, both the standard deviations for the 3 replicas in the OX state and 3 replicas in the NSQ state were relatively large (Figure 5A), giving a total standard deviation of 113 mV for the redox potential. This is due to a too-small phase space overlap between adjacent λ windows; the BAR relative entropy diagnostics (*s*_A_ and *s*_B_) would frequently exceed a value of 2 and even reach values as high as 6. Therefore, in the remainder of the tests carried out in this system, we use 40 λ windows, which reduced the *s*_A_ and *s*_B_ diagnostics to values that are usually smaller than 1 (and no larger than 1.7). We did not test the effect of going to a larger number of λ windows, since most free energy protocols applied to similarly sized systems employ fewer than 40 λ windows.^112,152^

The next decision is related to the length of the equilibration and production runs. For each λ window, we equilibrate the system for 3 ns, followed by a 12 ns production run. The choice of 12 ns is motivated by earlier studies on flavodoxins; for example, Sattelle and Sutcliffe used thermodynamic integration with 14 discrete λ windows each 1.5 ns long, while Silvestri et al. used a total simulation time ranging from 10 to 500 ns (for the entire simulation).^112,113^ Benchmark studies on other systems by Michel and co-workers also indicated convergence of absolute binding free energies with 6 ns per λ window, even though their simulations involved binding of a large molecule to a protein.^153^ Combined, our simulations are 15 ns per λ window (600 ns in total for 40 λs). Since the redox potential is computed from 3 replicas in the OX state and 3 replicas in the NSQ state, that means that each redox potential is the output of 3.6 μs of simulation time. Given the relatively small size of *Desulfovibrio vulgaris* flavodoxin (147 amino acids) and the size-conserving mutation (D63N), these simulation lengths should be adequate.

Most of the tests in this work were performed using 3 ns equilibration, but we also tested the effect of increasing the initial equilibration (only at λ = 0) from 3 to 50 ns. Since we employ serial equilibration, increasing the initial equilibration time also benefits the equilibration for subsequent steps. The result, as discussed in more detail below, is that when the system is properly described using a reasonable ionic strength, then there is a limited benefit to increasing the length of the initial equilibration.

### The Effect of the Solution’s Ionic Strength

As shown in Figure 3A, flavodoxin is a very negatively charged protein. Here, we compare models using a minimal NaCl concentration (i.e., just 20 Na^+^ ions to neutralize the system) versus the 100 mM extra NaCl model (which includes 42 additional NaCl ion pairs). Figure 5B shows a clear reduction in standard deviations when moving from a minimal NaCl model to the extra NaCl model.

To better understand the source of this error, we show in Figure 6 the distribution of solution ions (Na^+^ and Cl^–^) in the region surrounding the mutation site. Specifically, we plot the coordination number (CN), a metric that provides the average number of Na^+^ (and/or Cl^–^) ions around the OD2 atom of D63. This is the atom that is transformed into a nitrogen during the course of the D63N alchemical perturbation (see Figure 3B). Figure 6A shows 3D surface plots of the Na^+^ CN as a function of distance from the reference OD2 atom and as a function of λ. A clear trend observed in both the minimal and the extra NaCl models is a decrease in Na^+^ coordination as λ progressively switches between 0 (ASP/D63 state) and 1 (ASN/N63 state). This can be anticipated due to the elimination of the negative charge of the aspartic acid during the course of the free energy simulations.

**Figure 6:**
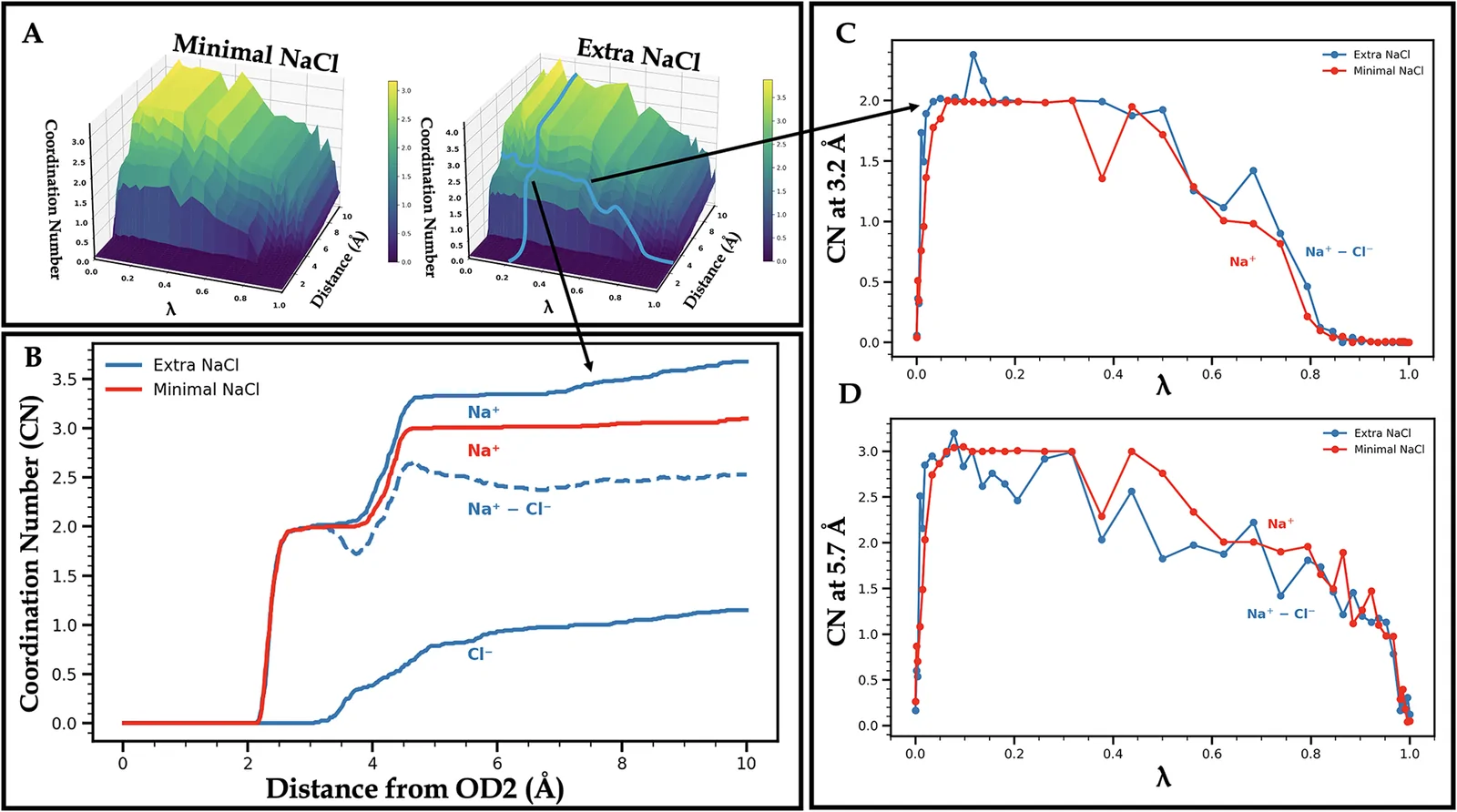
Effect of minimal vs extra NaCl solution ions on the electrostatic environment near the alchemically transformed D63N oxygen (labeled OD2, see Figure 3B). (A) 3D surface plots showing the coordination number (CN, i.e., the integrated radial distribution function) of Na^+^ ions as a function of λ and distance from OD2. The plots are shown for a representative replica of the OX state from a model containing minimal counterions and another model containing extra (42 pairs) of NaCl ions. (B) The CN of sodium and chloride ions as a function of distance to OD2 shown for λ = 0.20634 (the 16th λ window). Specifically, for the minimal NaCl model we show the Na^+^ distribution around OD2 (in red), while for the extra NaCl model we show in blue the Na^+^, Cl^–^, and the charge CN calculated as Na^+^ – Cl^–^. (C, D**)** The Na^+^ CN for the minimal model and the charge (Na^+^ – Cl^–^) CN for the extra NaCl model as a function of λ at 3.2 Å and 5.7 Å from OD2, respectively.

Figure 6B is a “slice” or cross section of the 3D plot at λ = 0.20634, where the D63 side chain is approximately 20% through its transformation to N63. Specifically, the panel represents the CN as a function of distance from OD2. A clear difference emerges between the minimal and extra NaCl models here. In the minimal model, there are no chloride ions in solution and therefore only the Na^+^ CN is shown in red. On average, there are two Na^+^ ions that remain close to the D63 at a distance of around 2 Å. This distance is comparable to the combined van der Waals radii of the Na^+^ and OD2 atoms, suggesting that the ions stay paired as closely as possible throughout the simulation window. At a distance of 4 Å, a third Na^+^ ion appears, likely the one that is paired with the adjacent negatively charged D62 amino acid. This gives rise to different Na^+^ “ionic shells” surrounding OD2: the first shell is at around 2 Å, corresponding to the first plateau observed in red in Figure 6B. The second shell is associated with the nearby D62.

The extra NaCl model behaves similarly to the minimal model up to 3 Å. In other words, 2 Na^+^ are also paired with OD2 and remain close throughout the simulation window. However, mainly due to the presence of Cl^–^ in solution, differences emerge in the electrostatics after 3 Å where the Cl^–^ CN begins to rise. This is qualitatively consistent with a Debye–Hückel model in which each charge is surrounded by nearby opposite charges.^154^ In Figure 6B, we plot the difference in CN between Na^+^ and Cl^–^ shown as a blue dashed line representing the total integrated charge within each distance. The appearance of Cl^–^ at 3 Å causes a “dip” in the charge lasting up to 4 Å. Beyond that distance, the second ionic shell starts. However, here, the Na^+^ CN increases to a value of 3.25 for the extra NaCl model instead of 3 (compare the blue and red Na^+^ CN curves). The presence of a chloride shell in the extra NaCl model therefore attracts an additional Na^+^ ion which spends some of its time (approximately 25% of this simulation) in the second ion shell starting at 4 Å. When we take the difference between the Na^+^ and Cl^–^ (dashed blue line) we find even more pronounced deviation from the red (minimal ion) curve, indicating a very different charge profile in the second ionic shell compared to the first around the mutant. Importantly, it is in this second ionic shell where flavin resides, and therefore these differences in *medium range* electrostatics will affect the redox potential.

In Figure 6C,D, we show a different cross section of the 3D plots by plotting the CN as a function of λ at two specific distances. Here, the CN values shown in red belong fully to the Na^+^ CN for the minimal NaCl model (since there are no Cl^–^ ions), while the CN values shown in blue are the difference between Na^+^ and Cl^–^ CNs (i.e., Na^+^ – Cl^–^) in the extra NaCl model. Therefore, these CNs represent the total integrated charge originating from the solution ions in both models. Panel C represents the ion distribution in the first ionic shell (∼ 3.2 Å), while panel D represents the ion distribution for the second ionic shell (∼ 5.7 Å). The values of 3.2 Å and 5.7 Å are chosen from the middle of the two plateaus of plot in panel B. The middle of the plateau was chosen to increase our confidence that we are capturing the plateau region for other λ steps rather than selecting a value close to the edge of the plateau where fluctuations occur from one λ step to the other.

A minor but possibly relevant detail here is the sudden rise in integrated charge at λ = 0 to around λ = 0.05 in panels C and D of Figure 6. We speculate that this sudden increase is due to the breaking in charge symmetry of the D63 side chain as it is converted to asparagine (see Figure 3B). In D63, the charge on both oxygen atoms is equal (−0.8), and therefore the simulations sample their positions interchangeably. Once one of the aspartate oxygen atom starts to morph into an NH_2_ group, this locks the side chain into a specific orientation with a specific surrounding ionic distribution.

Comparison of the red and blue curves in panel C indicates a largely consistent charge behavior as a function of λ. Therefore, *short-range* electrostatics appear to be described well by either model (minimal or extra NaCl). Since short-range electrostatics contribute most significantly to energetics, capturing those correctly is important for quantitative free energy calculations in general. This helps explain the success of some protocols using minimal counterions in binding free energy simulations.

The second ionic shell (panel D) captures differences between the extra and minimal NaCl models across a wide range of λ values, not just at λ = 0.20634. While the 5.7 Å distance is further away from the OD2 of D63 mutant, these charge differences are closer to FMN and therefore affect the description of electrostatics in the region between the mutation and the redox cofactor, which is critical for the redox simulations.

### Treatment of the Counterion

The D63N mutation introduces a net positive charge in the system due to the transformation of a negatively charged residue to a neutral one. Beyond the effect of using minimal or extra solution ions, it is useful to compare different approaches to achieve charge neutrality during the course of the λ transformation. As mentioned in the Methods section, we test a number of different approaches termed NAB, BCL, and PME. The NAB/BCL approaches involve an alchemical transformation that eliminates/creates a Na^+^/Cl^–^ counterion as a function of λ. This counterion (NAB or BCL) may be flexible to move or fixed at a point far from the protein and close to the solvent box edge. PME, on the other hand, neutralizes the growing positive charge from D63N by gradually introducing a uniform neutralizing negative background charge throughout the model.

Figures 4 and 5 indicate that the NAB approach typically results in the lowest standard deviation and gives a redox potential shift more consistent with the experiment. The PME approach appears to perform second best, giving a smaller redox potential shift with a slightly larger standard deviation. Finally, the BCL approach performs worse, either giving large systematic errors or large random errors, depending on whether the BCL is fixed or not.

To explain these trends, Figure 7A,B present similar analyses as those in Figure 6B,D, respectively. Specifically, Figure 7A,B displays the total “ion difference” CN as a function of distance, calculated at a fixed λ = 0.20634, while plots the same CN values but as a function of λ at a fixed distance of 5.7 Å. To demonstrate that the analyses are statistically significant and not random, these plots are derived from an average of three replicas. For panel A, we include the results for flavin in the NSQ redox state, in addition to the OX state. Differences in the environments around these two redox states can help explain systematic errors.

**Figure 7:**
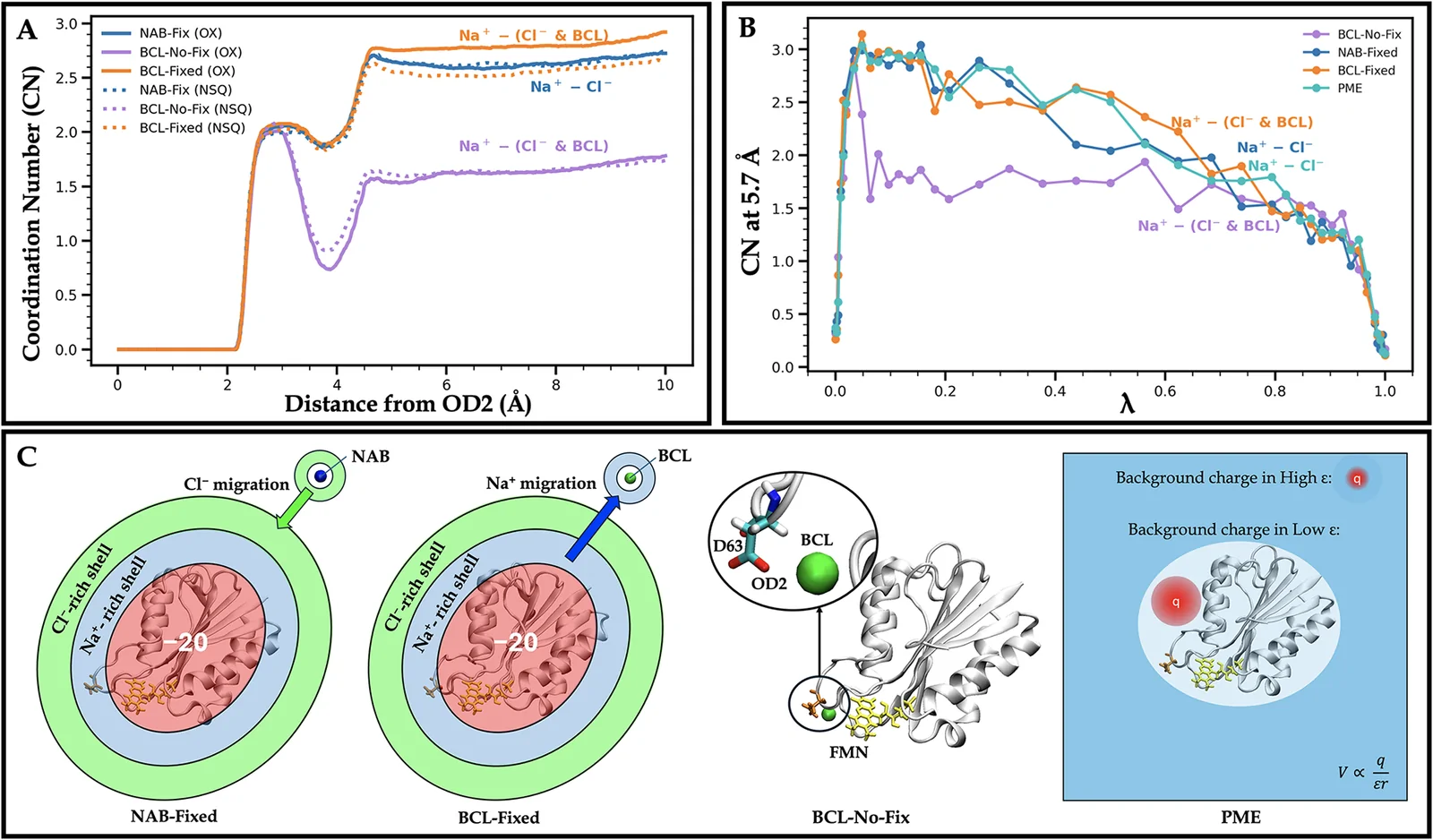
Effect of using different charge balancing schemes in the alchemical simulations. (A) The CN of solution ions as a function of distance to OD2 shown for λ = 0.20634 (the 16th λ window). This plot is generated from an average of 3 replicas for each of the OX and NSQ states. In the NAB models we show in blue the charge CN calculated as Na^+^ – Cl^–^. However, for BCL, we show in orange (for the fixed BCL model) and in lilac (for nonfixed BCL model) the Na^+^ – (Cl^–^ and BCL) ions. Note that, for the BCL nonfixed model this does not correspond to a true integrated charge, since BCL has a fractional charge at λ = 0.20634 rather than a full negative charge. (B) The (Na^+^ – Cl^–^) for the NAB and PME background charge methods and Na^+^ – (Cl^–^ and BCL) for the fixed and nonfixed BCL models as a function of λ at 5.7 Å from OD2, respectively. These plots are generated from the average of 3 replicas for the OX state only, since the NSQ state gave qualitatively similar plots. (C) Schematic representations to explain the sources of random and systematic errors in each of the counterion charge balancing approaches. See text for more details. All the data in this figure uses the extra NaCl model with GROMACS 2024.4.

First, it is worth comparing the NAB vs BCL approaches, where both counterions are *fixed*. Although they are fixed far from the protein or active site, the two protocols are not equivalent. In the NAB approach, one positive and one negative charge vanish together *at a fixed distance* for both the OX and NSQ states. Recall that the FMN is also kept frozen in this simulation, therefore pinning the D63 amino acid nearby. The screened electrostatic interaction between those distant charges will cancel out in two legs of the thermodynamic cycle in Figure 2 and therefore will not contribute to the systematic error. On the other hand, the BCL-fixed scenario is associated with a negative charge disappearing at the mutation site and reappearing near the edge of the box. This effectively creates a charge migration toward BCL. The two images on the left in Figure 7C illustrate why this can yield larger errors in the redox simulations. Introducing the negatively charged BCL ion will attract the positively charged Na^+^ ions. Those ions will migrate partly from free Na^+^ ions in solution, but also from the Na^+^-rich shell surrounding the protein, resulting in a higher Na^+^ concentration in solution (compare the orange BCL line in Figure 7A to the blue line). For finite simulation times, the distribution of Na^+^ ions between the Na^+^-rich region near the protein, the free Na^+^ ions in solution, and the newly formed Na^+^-rich shell around BCL may remain out of equilibrium.^155,156^ Since Na^+^ ions are critical to neutralize the very negative (−20) flavodoxin protein charge, a charge migration away from this Na^+^-rich shell will affect the protein dynamics and energetics. These effects will be exacerbated in the minimal counterion approach, and can certainly help explain the larger standard deviations observed there as well.

The NAB protocol also involves charge migration away from the positively charged NAB ion as it is annihilated. However, the Cl^–^ ions released from the solvation shell around NAB go back to solution and mainly toward the *second* ionic shell around the protein, which will have a smaller perturbative effect on the system’s energy.

Given the above discussion, it may be a surprise that BCL-nonfixed approach has a small standard deviation (47 mV, which is even smaller than the standard deviation of the NAB-fixed protocol). However, the BCL-nonfixed approach introduces a large systematic error, giving a redox potential shift of −324 mV instead of the experimental redox potential of −189 mV. This systematic error can be understood as a result of soft-core potentials for the alchemically altered species. When two entities (here, D63 and BCL) with soft-core potentials interact, they are more likely to settle into a shared energy minimum.^157^ Indeed, Figure 7A indicates a large reduction in the ion difference CN for the BCL-nonfixed protocol, which can be attributed to BCL’s presence in close proximity to the D63 (see the third image in Figure 7C). Figure 7B indicates that BCL remains close to the mutation site throughout almost all λ windows, at least up to λ = 0.8, resulting in an altered charge profile. This clearly indicates that BCL disproportionately (compared to other regular Cl^–^ ions) prefers to remain close to D63 due to their soft-core interaction. Importantly, this short-range interaction (between BCL and D63 mutant) potentially contributes a significant energy to the system. It also appears to be affected by the redox state of nearby flavin (compare the dashed and solid lilac lines in Figure 7A), especially at short-range near 4 Å where other models behave more consistently with each other). This difference can explain the systematic error observed for the BCL nonfixed protocol. In general, the comparison of the fixed and nonfixed models indicates that a fixed coalchemical ion approach is more appropriate to avoid the interactions between entities described with the soft-core potential.^140^

Unlike NAB and BCL, the PME approach simply introduces a uniform background negative charge to counter the disappearing D63 negative charge. Although this protocol appears to reproduce the experimental reduction potential accurately, it gives a slightly larger standard deviation compared to the NAB-fixed protocol. To understand the limitations of this model, consider the right-most image in Figure 7C. Due to the inverse relationship between the electrostatic potential and dielectric constant, a negative background charge introduced in a higher dielectric medium (e.g., a polar solvent like water) will be more strongly screened, which attenuates its electrostatic interactions. Instead, the same uniform negative background charge, when introduced in a lower dielectric environment like that of the protein, will have a more pronounced effect.^158^ This biases the simulation so that positively charged species become more attracted to the lower dielectric environment as λ increases. Indeed, on average, we find that the PME simulation maintains a more positive charge in the vicinity of OD2 than most other models throughout the λ range from 0 to 1 in Figure 7B (cyan plot). Of course, the effect is very subtle, which is why this method compares favorably to the BCL protocol.

### Effect of Accounting for the Kinetic Energy Contribution from Mass Perturbation

As atoms are alchemically transformed during the λ = 0 → 1 transition, their masses are also morphed along with their charge and Lennard-Jones parameters. In early versions of GROMACS (2024.3 and earlier), the kinetic energy contribution of such a mass morphing was not included in *gmx bar*.

Although masses do not affect the total AMBER parameter potential energy function, they do affect the acceleration of atoms during the Verlet algorithm. In other words, a heavier atom will accelerate more slowly when energy is transferred to it compared to a lighter atom. These effects are expected to be subtle when heavy atoms morph into each other (e.g., a morphing of oxygen to nitrogen in D63N). However, the dummy atoms, which gain mass from zero, can be more strongly affected. This is illustrated in Figure 8, where we ran a test by releasing the constraints on hydrogen atoms that were enforced during the LINCS algorithm and output the structure every 0.5 fs (using a 0.5 time step). In the top panel, at λ = 0, the energy rapidly becomes distributed to the massless dummy atoms, which start to oscillate just femtoseconds after being released. Instead, at λ = 1 where those dummy atoms are transformed into H atoms with mass 1, they now equilibrate more slowly, showing smaller oscillations during the early dynamics compared to the same atoms at λ = 0. This represents the correct behavior of this system; indeed, as the H atoms gain mass, they should accelerate more slowly as energy is distributed to them.

**Figure 8:**
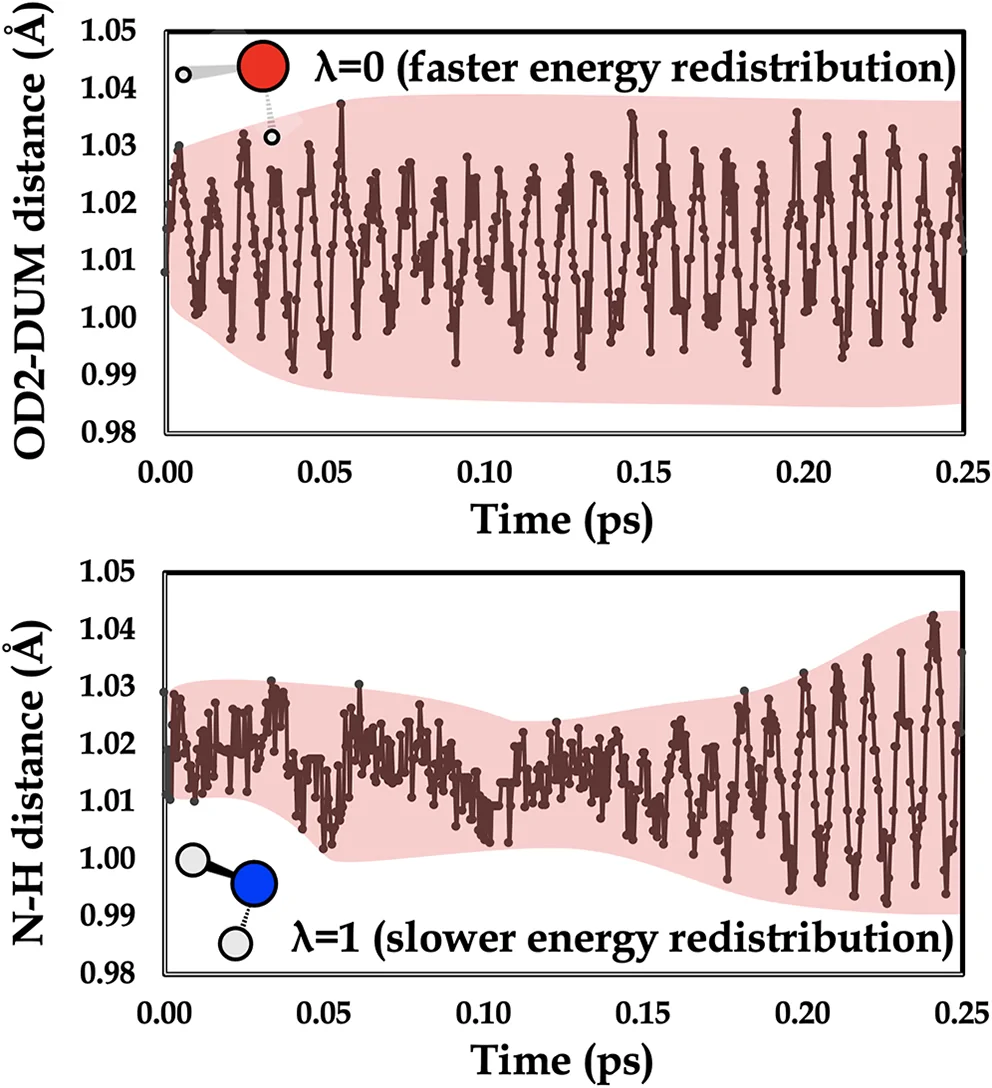
Distance between the OD2 and dummy H atoms at λ = 0 and at λ = 1 for a test carried out with a time step of 0.5 fs and releasing the H-bond constraints from LINCS. At λ = 1, the transition from the OD2 and massless dummy atoms to N and H atoms is complete, hence the change in the *y*-axis label.

The test above simply demonstrates that mass morphing does contribute to the behavior and energetics of the system. It is difficult to pinpoint exactly how missing contributions from mass morphing may contribute to random and systematic errors in redox potentials, but it can be expected that trajectories where the D63N side chain samples a larger number of configurations will have a different kinetic energy contribution than one where it is more static. The mobility of D63N may also be affected by the redox state of the nearby FMN. Therefore, such kinetic energy contributions can contribute to the larger random and systematic error observed with GROMACS 2024.2 (−244 ± 110 mV, despite using the best extra NaCl/NAB model) compared to 2024.4 (−203 ± 55 mV, see Figures 4 and 5). The same trend in errors is replicated in a second test for 2024.2 and 2024.4 using a minimal NaCl model (around the middle of Figure 4).

The effect of this mass perturbation may be relatively subtle here for D63N, which involves a relatively small change in mass. We expect that accounting for the mass change would be more important for a larger perturbation such as a mutation of a small amino acid to a large one (e.g., D to W).

## Conclusions

For classical free energy simulations, the 1 kcal/mol chemical accuracy value (equivalent to 43 mV) is widely cited as a desirable and obtainable level of accuracy. Here, we have not attempted to compute absolute redox potentials (ARPs) using binding free energies, but instead focused on computing relative redox potentials (RRPs) associated with a single mutation, D63N. Although this mutation conserves the size of the amino acid side chain, it annihilates a negative charge from the system, which requires proper treatment. We view such testing as an important preliminary step before carrying out more systematic studies on a series of different mutations.

It is understood that a well-parametrized force field and proper sampling are important considerations when it comes to classical free energy simulations. Here, we employ a hybrid APEC-RRP approach where QM/MM structures generated by APEC-F 2.0 are used as starting points for running alchemical free energy simulations associated with mutating an amino acid side chain. During the free energy simulations, the FMN is kept frozen in its QM/MM optimized geometry while the rest of the protein, solvent, and solution ions are sampled by molecular dynamics. The approximations used in this APEC-RRP approach are suitable for flavin due to the rigidity of its redox-active π-conjugated isoalloxazine moiety, which does not sample conformations other than minor methyl group rotations.

In addition to testing the APEC-RRP approach, the main objective of this work was to probe different factors related to the model setup, such as the inclusion of salt ions and the treatment of the counterion. While such model details may be less emphasized in computational redox studies, they can play an important role in determining the accuracy of the free energy simulations.

We find that indeed an error on the order of 1 kcal/mol is attainable for each branch of the thermodynamic cycle used to compute RRPs. However, the total error for introducing a charged mutation is at least 55 mV (1.27 kcal/mol) in the best model. Importantly, in other models where even apparently minor details are different, such as using a minimal salt concentration or using a different counterion scheme, the errors encountered can rapidly increase to several kcal/mol. We discussed the source of these errors for charged mutations and presented best practices for dealing with a charged mutation for RRPs. Specifically, using a coalchemical counterion fixed far away from the mutation site and having the opposite charge to the mutant appears to work best. We also emphasize that having additional solution ions, beyond a minimal counterion concentration, is essential to maintain realistic ionic environments surrounding the mutation. Although short-range and long-range electrostatics are consistent in the minimal and extra NaCl models, differences are found mainly in the medium range, around the second ionic/solvation shell. These medium-range electrostatics affect the environment and energetics of other nearby interactions.

This work focused on a solvent-exposed size-conserving mutation, D63N. On the one hand, exposure of this mutation site to the solvent and solution ions makes it more sensitive to details of the model such as ionic strength and counterion treatment. However, free energy simulations of buried amino acids are plagued by a different set of challenges. In a recent study on the measurement and control of redox potentials in iLOV,^67^ an internal amino acid (Q103) facing FMN’s N5 atom was mutated to different amino acids (A, G, R, K, E, and D). Free energy simulations were carried out in that work following exactly the same “best” protocol used here, using extra NaCl and coalchemical approach introducing an oppositely charged counterion (in that case, BCL was used to neutralize the growing positive charge when Q was mutated to K or R). However, larger errors were observed for this computational protocol, mainly due to two factors: first, several mutations involved large changes in amino acid size, and second, water molecules intermittently entered the binding site. Only by binning the free energy simulations into different categories (water-in replicas vs water-out replicas in the OX and NSQ states) did we obtain free energy results having an accuracy comparable to those reported here.^67^

Overall, there are clearly several remaining challenges associated with computing redox free energies, especially for systems involving a change in charge of either the redox cofactor or of the surrounding amino acids. As discussed in the Introduction and references therein, there are ongoing efforts to improve the accuracy of protein redox predictions through, for instance, enhanced sampling techniques or more accurate polarizable force fields. Enhanced sampling methods may include using algorithms such as accelerated molecular dynamics to better sample the conformational space of the protein and reach an equilibrium ion distribution more efficiently, or allow for the calculation of additional λ windows at a reduced computational cost.^159,160^ Polarizable force fields, on the other hand, better capture the polar and charged interactions between the flavin, the mutated amino acid, and the remaining environment, giving a more accurate physical description of those interactions that may be important when charged residues are involved.^4,103,110^ Recent developments, such as machine-learned potentials, may deliver both enhanced sampling and more accurate potentials at the same time.^161,162^ However, this work also emphasizes the importance of correctly describing the ionic environment to capture medium-range electrostatics accurately.

## Supplementary Material





## Data Availability

The data underlying this study are available in the published article, the Supporting Information, at 10.5281/zenodo.20518683 and on github https://github.com/gozem-gsu/Automated-APEC-F. The scripts used to generate and analyze the results are available at https://doi.org/10.5281/zenodo.20518684. This includes QM/MM optimized pdb and gro structures for the minimal and extra NaCl OX and NSQ models, hybrid topology files, sample mdp files, and a script that modifies the gro, top and itp files to alter the labels of dummy hydrogens. APEC-F 2.0 QMMM code is available on github: https://github.com/gozem-gsu/Automated-APEC-F. The associated user’s manual includes a more detailed step-by-step description of the protocol, as well as instructions for how to install the necessary software and set up the code, available at this link: http://gozemlab.com/APEC-F-2.0-Manual.pdfhttp://gozemlab.com/APEC-F-2.0-Manual.pdf. A video tutorial to get started with the running the automated APEC-F 2.0 is available at this link: https://www.youtube.com/watch?v=QKPV2fbTPbQ.

## Notes

Elaboration on the effect of mass morphing in gmx bar output and table of Δ*G* from each of 3 replicas of OX and NSQ states for each protocol tested (PDF)
Decomposition of computed Δ*G* from 3 replicas of OX and NSQ states and their standard deviations with the average Δ*G* for OX and NSQ and average standard deviation, the computed *E*_red_ and its standard deviation for each model with varying conditions is tabulated in the last two columns (Table S1) (XLSX)
