# Supplementary material for: Toward Relative Redox Potential Predictions in Flavoproteins: Treatment of a Charge-Changing Mutation in Alchemical Free Energy Simulations
Source: J Chem Theory Comput. 2026 Aug 13;22(16):8682–99. doi: 10.1021/acs.jctc.6c01142 (PMC13523640; doi:10.1021/acs.jctc.6c01142)
Supplement: Supplementary file 1 [file ct6c01142_si_001.pdf]

Supporting Information

Towards relative redox potential predictions in flavoproteins: Treatment of a charge-changing mutation in alchemical free energy simulations

Sarah Elhajj[†], Jacopo D'Aszensi[‡], Massimo Olivucci[‡,¶], Samer Gozem*,[†]

[†] Department of Chemistry, Georgia State University, Atlanta, Georgia, 30302, United States
sgozem@gsu.edu

[‡] Dipartimento di Biotecnologie, Chimica e Farmacia, Universita di Siena, Via A. Moro 2, 53100 Siena, Italy

[¶] Chemistry Department, Bowling Green State University, Bowling Green, Ohio 43403, United States

S1 Elaboration on the effect of mass morphing in gmx bar output for versions released before gromacs 2024.4

Here we elaborate more on the effect of mass morphing during a mutation, such as converting an aspartate (**D**) to an asparagine (**N**). In this case, the mass of the involved atoms morph as the system transitions between states. To compute the free energy differences, we consider here for the purpose of this supporting section the effect of mass morphing in Thermodynamic Integration (TI), an analogous but different approach compared to the Bennett Acceptance Ratio (BAR) method used in the main manuscript.

In TI, at each λ state (where a corresponding molecular dynamics (MD) simulation is run), if the `mdp` file includes the free energy section, GROMACS computes, for each frame, the derivative of the system's total energy (kinetic + potential) with respect to lambda. λ represents the coupling parameter, and the difference between consecutive λ values determine the incremental change for the next MD simulation.

For any given frame in an MD simulation at a particular λ, GROMACS calculates the energy difference for that specific conformation when transitioning to the next λ value. This energy difference forms the numerator of the derivative. While this process is complicated by the fact that conformations differ between states due to residue morphing, we can simplify the explanation by imagining that an atom of **D** coincides with an atom of **N** in the current frame. For example, an oxygen (**O**) atom in **D** may correspond to a nitrogen (**N**) atom in **N**.

The computed quantity includes a kinetic energy contribution, ΔKinEn, given by:

$$\Delta \text{KinEn} = \frac{1}{2}\Delta m_j \cdot v_j^2$$

Here:

- Δm_j is the mass difference for the j-th atom between the two states (e.g., the mass of an oxygen minus the mass of a nitrogen).
- v_j is the velocity of the atom in the current frame.

Neglecting the Δm_j term would nullify ΔKinEn, which should be included as part of the total energy difference (the numerator of the derivative).

The effect can be understood by looking at the equation for ΔA in the TI framework:

$$\Delta A = \sum_i \left\langle \frac{\partial H}{\partial \lambda} \right\rangle_i (\Delta\lambda)_i$$

Here, the summation runs over the total number of λ windows, $\left\langle \frac{\partial H}{\partial \lambda} \right\rangle$ is the ensemble average of the energy derivative at a given λ at which a corresponding MD trajectory is run, and $(\Delta\lambda)_i$ is the incremental change in λ in the i-th λ window.

Table S1: Table showing the decomposition of computed ΔG from 3 replicas of OX and NSQ states and their standard deviations with the average ΔG for OX and NSQ and average standard deviation. The computed E_{red} and its standard deviation for each model with varying conditions is tabulated in the last two columns.

λ steps	NVT0 (ns)	Salt?	Countercharge	Ion Fixed?	2024.4	OX ΔG_1 kcal/mol	OX ΔG_2 kcal/mol	OX ΔG_3 kcal/mol	NSQ ΔG_1 kcal/mol	NSQ ΔG_2 kcal/mol	NSQ ΔG_3 kcal/mol	$\langle\Delta G\rangle$ OX kcal/mol	$\langle\Delta G\rangle$ NSQ kcal/mol	$\sigma_{\Delta G}$ OX kcal/mol	$\sigma_{\Delta G}$ NSQ kcal/mol	E_{red} (mV)	$\sigma_{E_{\text{red}}}$ mV
20	3	min	NAB	y	y	319.09	327.94	314.88	334.02	319.58	319.4	76.6	77.52	1.6	2.0	-186	111
20	3	extra	NAB	y	y	319.12	309.2	315.52	303.8	313.31	311.84	75.2	74.01	1.2	1.2	-97	74
20	50	min	NAB	y	y	295.34	306.58	308.2	307.86	312.12	326.36	72.5	75.39	1.7	2.3	-273	124
20	50	extra	NAB	y	y	314.93	307.62	325.98	305.08	312.89	316.19	75.6	74.42	2.2	1.4	-98	113
40	3	min	BCL	y	y	-338.59	-348.57	-324.21	-317.75	-330.62	-337.92	-82.1	-78.58	1.2	2.4	-300	118
40	3	min	NAB	y	n	302.11	308.36	305.94	334.14	319.5	303.13	73.0	76.22	0.8	3.7	-287	164
40	3	min	NAB	y	y	308.95	311.19	311.69	321.65	310.26	302.33	74.2	74.43	0.3	2.3	-156	102
40	3	extra	BCL	n	y	-344.91	-347.06	-341.08	-326.3	-331.16	-324.52	-82.3	-78.23	0.7	0.8	-324	47
40	3	extra	BCL	y	y	-346.31	-350.91	-346.02	-341.98	-355.22	-354.45	-83.1	-83.78	0.7	1.8	-119	82
40	3	extra	PME	y	y	-11.78	-20.08	-17.25	-21.17	-11.31	-13.2	-3.9	-3.64	1.0	1.3	-160	70
40	3	extra	NAB	n	y	312.44	320.56	326.39	314.73	317.08	316.21	76.4	75.53	1.7	0.3	-109	74
40	3	extra	NAB	y	n	311.03	304.77	290.33	310.94	310.89	312.19	72.2	74.41	2.5	0.2	-244	110
40	3	extra	NAB	y	y	305.06	315.05	308.09	313.34	315.95	314.89	73.9	75.22	1.2	0.3	-203	55
40	50	extra	NAB	y	y	305.28	314.72	305.8	316.07	310.65	308.21	73.8	74.48	1.3	1.0	-180	69
